# Mechanosensory Signaling in Astrocytes

**DOI:** 10.1523/JNEUROSCI.1249-20.2020

**Published:** 2020-12-02

**Authors:** Egor A. Turovsky, Alice Braga, Yichao Yu, Noemi Esteras, Alla Korsak, Shefeeq M. Theparambil, Anna Hadjihambi, Patrick S. Hosford, Anja G. Teschemacher, Nephtali Marina, Mark F. Lythgoe, Philip G. Haydon, Alexander V. Gourine

**Affiliations:** ^1^Centre for Cardiovascular and Metabolic Neuroscience, Department of Neuroscience, Physiology & Pharmacology, University College London, London WC1E 6BT, United Kingdom; ^2^Department of Neuroscience, Tufts Neuroscience Institute, Tufts University School of Medicine, Boston, Massachusetts 02111; ^3^UCL Centre for Advanced Biomedical Imaging, Division of Medicine, University College London, London WC1E 6DD, United Kingdom; ^4^Department of Clinical and Movement Neurosciences, UCL Queen Square Institute of Neurology, London WC1N 3BG, United Kingdom; ^5^Department of Biomedical Sciences, University of Lausanne, Lausanne 1005, Switzerland; ^6^Physiology, Pharmacology, and Neuroscience, University of Bristol, Bristol BS8 1TD, United Kingdom

**Keywords:** astrocytes, baroreceptor, cardiovascular, glia, mechanosensory, sympathetic

## Abstract

Mechanosensitivity is a well-known feature of astrocytes, however, its underlying mechanisms and functional significance remain unclear. There is evidence that astrocytes are acutely sensitive to decreases in cerebral perfusion pressure and may function as intracranial baroreceptors, tuned to monitor brain blood flow. This study investigated the mechanosensory signaling in brainstem astrocytes, as these cells reside alongside the cardiovascular control circuits and mediate increases in blood pressure and heart rate induced by falls in brain perfusion. It was found that mechanical stimulation-evoked Ca^2+^ responses in astrocytes of the rat brainstem were blocked by (1) antagonists of connexin channels, connexin 43 (Cx43) blocking peptide Gap26, or Cx43 gene knock-down; (2) antagonists of TRPV4 channels; (3) antagonist of P2Y_1_ receptors for ATP; and (4) inhibitors of phospholipase C or IP3 receptors. Proximity ligation assay demonstrated interaction between TRPV4 and Cx43 channels in astrocytes. Dye loading experiments showed that mechanical stimulation increased open probability of carboxyfluorescein-permeable membrane channels. These data suggest that mechanosensory Ca^2+^ responses in astrocytes are mediated by interaction between TRPV4 and Cx43 channels, leading to Cx43-mediated release of ATP which propagates/amplifies Ca^2+^ signals via P2Y_1_ receptors and Ca^2+^ recruitment from the intracellular stores. In astrocyte-specific Cx43 knock-out mice the magnitude of heart rate responses to acute increases in intracranial pressure was not affected by Cx43 deficiency. However, these animals displayed lower heart rates at different levels of cerebral perfusion, supporting the hypothesis of connexin hemichannel-mediated release of signaling molecules by astrocytes having an excitatory action on the CNS sympathetic control circuits.

**SIGNIFICANCE STATEMENT** There is evidence suggesting that astrocytes may function as intracranial baroreceptors that play an important role in the control of systemic and cerebral circulation. To function as intracranial baroreceptors, astrocytes must possess a specialized membrane mechanism that makes them exquisitely sensitive to mechanical stimuli. This study shows that opening of connexin 43 (Cx43) hemichannels leading to the release of ATP is the key central event underlying mechanosensory Ca^2+^ responses in astrocytes. This astroglial mechanism plays an important role in the autonomic control of heart rate. These data add to the growing body of evidence suggesting that astrocytes function as versatile surveyors of the CNS metabolic milieu, tuned to detect conditions of potential metabolic threat, such as hypoxia, hypercapnia, and reduced perfusion.

## Introduction

Systemic arterial blood pressure and heart rate are controlled by the neural circuits of the brainstem which continually fine-tune the autonomic vasomotor and cardiac nerve activities in accord with the prevailing physiological and behavioral needs. This intricate control relies on afferent information that is received by the brainstem autonomic control circuits from various sources. Baroreceptors located in the carotid bifurcation and the aortic arch are critically important for short-term (seconds and minutes) control of blood pressure and heart rate. In response to the increases in arterial blood pressure these stretch-sensitive baroreceptor neurons (Zeng et al., 2018), with projections in the aortic and carotid walls, initiate the arterial baroreflex resulting in a reduction in heart rate, cardiac contractility, and peripheral vascular resistance.

In addition to the inputs from the peripheral arterial baroreceptors, the activities of cardiovascular control circuits of the brainstem are strongly modulated by changes in cerebral perfusion pressure. Studies by Cushing (Cushing, 1901) and Rodbard and Stone (Rodbard and Stone, 1955) first suggested the existence of an intracranial baroreceptor that is activated by decreases in blood flow to the brain. Recently reported results of experimental studies performed in anaesthetized mice (Schmidt et al., 2018), rats (Marina et al., 2020), conscious sheep (Guild et al., 2018), and humans (Schmidt et al., 2018) confirmed the existence of an intrinsic brain mechanism capable of sensing physiological decreases in cerebral perfusion pressure. This mechanism triggers compensatory increases in systemic arterial blood pressure and heart rate to maintain cerebral blood flow, forming a homeostatic feedback loop.

There is evidence that astrocytes are the likely candidates for the role of intracranial baroreceptors (Marina et al., 2020). The end-feet of these numerous glial cells enwrap all penetrating and intraparenchymal cerebral blood vessels (Iadecola and Nedergaard, 2007), making astrocytes ideally positioned to sense changes in vascular lumen diameter and/or vascular wall stress associated with changes in flow (Kim et al., 2015). Astrocytes respond to acute decreases in cerebral perfusion pressure with immediate elevations in intracellular [Ca^2+^], while blockade of Ca^2+^-dependent signaling mechanisms in the astrocytes of the brainstem prevents compensatory increases in vasomotor and cardiac sympathetic nerve activities induced by reductions in brain perfusion (Marina et al., 2020).

To function as intracranial baroreceptors, astrocytes must possess a specialized membrane mechanism that makes them exquisitely sensitive to mechanical stimuli. Previous studies of mechanosensory signaling in optic nerve head and cortical astrocytes suggested potential involvement of mechanosensitive and stretch-activated channels, including pannexin 1 (Panx1) hemichannels (Beckel et al., 2014), transient receptor potential cation channel subfamily V member 4 (TRPV4) channels (Kim et al., 2015), and PIEZOs (Choi et al., 2015; Velasco-Estevez et al., 2020). In this study, we specifically investigated the mechanosensory signaling mechanisms leading to Ca^2+^ responses in brainstem astrocytes, because these cells reside within the cardiovascular sympathetic control circuits and mediate increases in blood pressure and heart rate in response to falls in cerebral blood flow (Marina et al., 2020).

## Materials and Methods

All experiments were performed in accordance with the European Commission Directive 2010/63/EU (European Convention for the Protection of Vertebrate Animals used for Experimental and Other Scientific Purposes) and the United Kingdom Home Office (Scientific Procedures) Act (1986) with project approval from the Institutional Animal Care and Use Committees of the University College London and Tufts University. The animals were group-housed and maintained on a 12/12 h light/dark cycle (lights on 7 A.M.) and had *ad libitum* access to water and food.

### 

#### Cell cultures

Primary cultures of brainstem astrocytes were prepared from the brains of rat pups (postnatal day 2–3 of either sex) as described in detail previously (Angelova et al., 2015; Turovsky et al., 2016). After isolation, the cells were plated on poly-D-lysine-coated coverslips and maintained at 37°C in a humidified atmosphere of 5% CO_2_ and 95% air for a minimum of 10 d before the experiments.

#### Recording changes in intracellular Ca^2+^

Optical measurements of changes in intracellular [Ca^2+^] in cultured astrocytes were performed using an inverted epifluorescence Olympus microscope, equipped with a cooled CCD camera (Retiga; QImaging) as described previously (Angelova et al., 2015; Turovsky et al., 2016). Changes in [Ca^2+^]_i_ were visualized using genetically encoded Ca^2+^ indicator *Case12* expressed in astrocytes using an adenoviral vector (AVV) with enhanced GFAP promoter (Gourine et al., 2010; Guo et al., 2010). The vector was added to the incubation medium on day 5 of cell culture preparation at ∼5 × 10^10^ transducing units ml^−1^. The specificity, Ca^2+^ sensitivity, and the dynamic range of *Case12* were described in detail previously (Gourine et al., 2010). The recordings were performed in a custom-made imaging chamber in HEPES-buffered solution (HBSS), containing the following: 156 mm NaCl, 3 mm KCl, 1 mm MgSO_4_, 1.25 mm КН_2_РO_4_, 2 mm CaCl_2_, 10 mm glucose, and 10 mm HEPES (рН 7.4) at ∼30°C. Changes in *Case12* fluorescence were monitored in individual cells using 490-nm excitation light provided by a xenon arc lamp (Cairn Research). Florescence emission was recorded at 530 nm.

#### Assessment of hemichannel open probability by dye loading

Connexin hemichannels are permeable to the fluorescent dye carboxyfluorescein (376 Da) and in an open state can act as conduits of carboxyfluorescein transport across the membrane in accord with the concentration gradient of the dye (Huckstepp et al., 2010; Meigh et al., 2013). Carboxyfluorescein (100 μm) was added to the cell incubation media for 10 min, resulting in background connexin‐mediated dye loading, followed by application of the experimental stimulus. Then the cells were washed for 5 min, and the degree of intracellular carboxyfluorescein accumulation (dye loading) was assessed by measuring the intensity of carboxyfluorescein fluorescence in individual cells. Images of carboxyfluorescein fluorescence were taken using Olympus FV1000 confocal microscope (Olympus) before and after addition of the dye, after the application of the mechanical stimulus in the presence of the dye in the media and after the washout of carboxyfluorescein. Using ImageJ software, regions of interest were drawn around the cell bodies of astrocytes and the mean pixel intensity for all the cells in the field of view was calculated. Background fluorescence was subtracted.

#### Mechanical stimulation of astrocytes in culture

**Figure 1.**
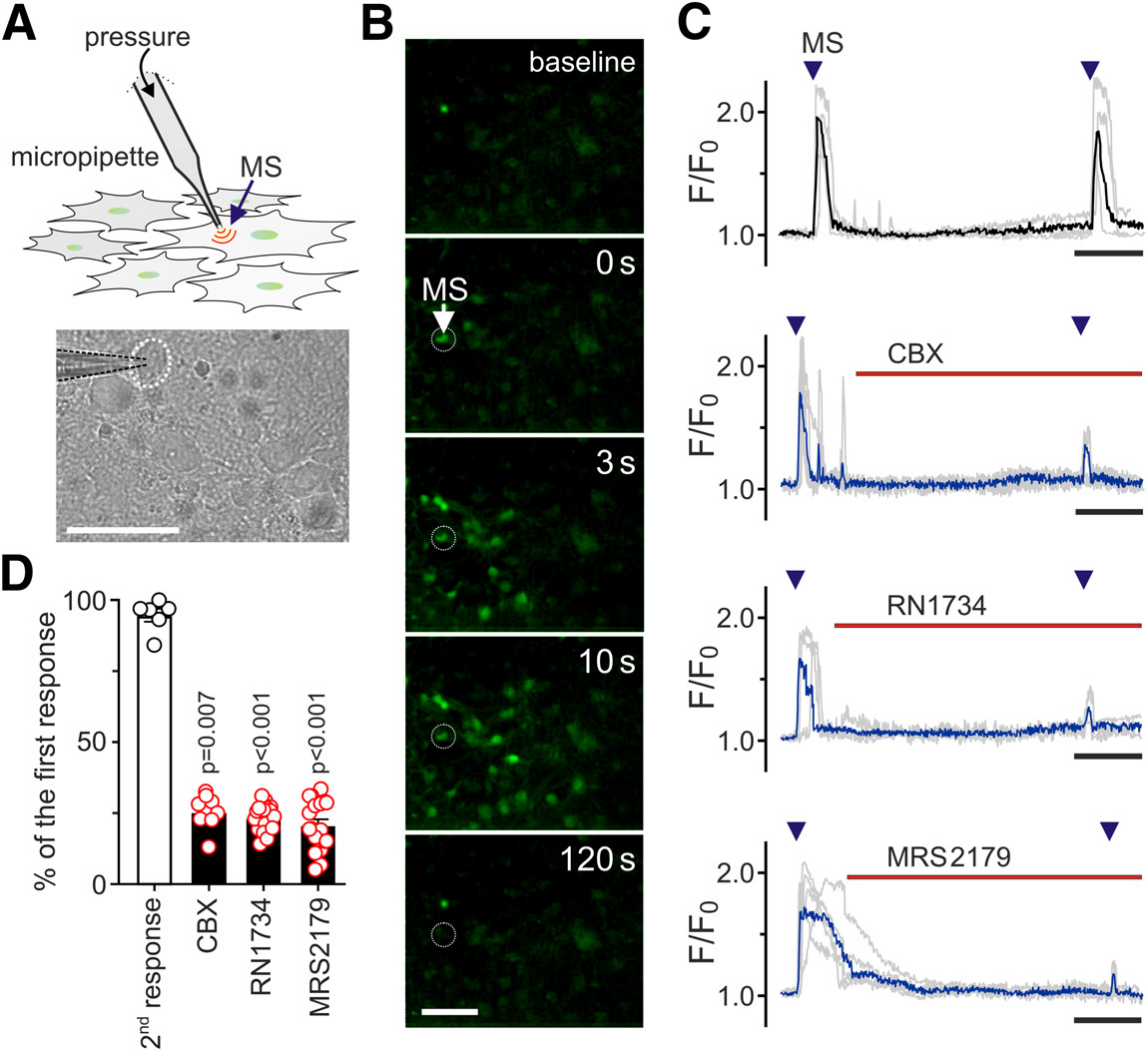
Mechanosensory Ca^2+^ responses in brainstem astrocytes. ***A***, Controlled mechanical stimulation (MS) of an individual astrocyte in culture applied by timed ejections of extracellular media by pressurization of a patch pipette positioned within 20–100 µm from the cell membrane. Scale bar: 50 µm. ***B***, Mechanical stimulation induced changes in fluorescence of Ca^2+^-sensitive genetically encoded sensor *Case12* expressed in cultured astrocytes. Representative images were taken at baseline and at the indicated times after mechanical stimulation of the cell indicated by the arrow. Scale bar: 100 µm. ***C***, Representative examples of mechanosensory [Ca^2+^]_i_ responses recorded in brainstem astrocytes in the absence and presence of connexin/pannexin channel blocker CBX (100 μm), TRPV4 channel inhibitor RN1734 (10 μm), or P2Y_1_ receptor antagonist MRS2179 (3 μm). Traces depict individual (gray) and averaged (black/blue) changes in fluorescence of Ca^2+^-sensitive *Case12*, recorded in three to five individual astrocytes in the same number of separate cultures in a single experimental session. Time bars: 150 s. ***D***, Summary data illustrating the effects of CBX, RN1734, and MRS2179 on mechanosensory Ca^2+^ responses in brainstem astrocytes. Data points show peak magnitude of the second [Ca^2+^]_i_ response (expressed as the percentage of the first response) recorded in individual cells in separate cultures (*n* = 6–20); *p* values, Kruskal–Wallis ANOVA.

**Figure 2.**
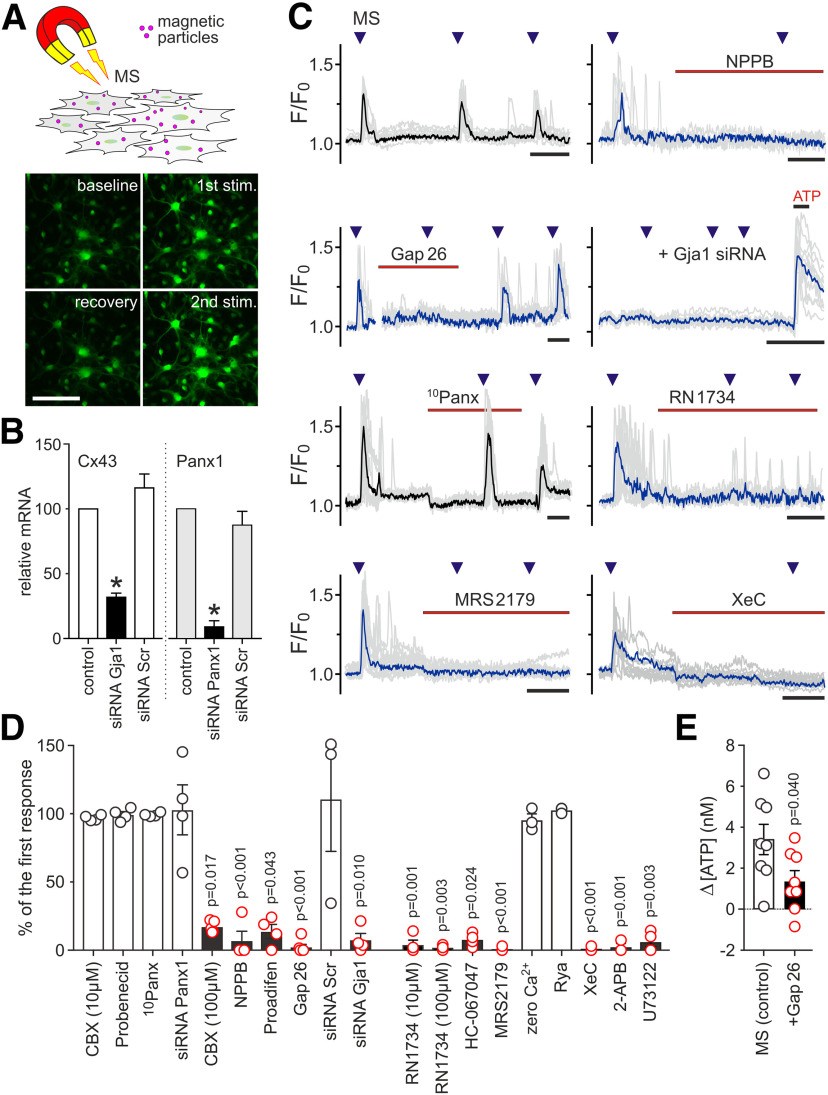
Cx43, TRPV4 channels, and P2Y_1_ receptors are critical components of mechanosensory Ca^2+^ signaling in astrocytes. ***A***, Mechanical en masse stimulation of astrocytes coated with magnetite particles by application of the magnetic field and representative images of *Case12* fluorescence in cultured astrocytes taken at baseline and at the peak of the response to repeated mechanical stimulation using this approach. Scale bar: 100 µm. ***B***, Summary data illustrating relative Cx43 (*Gja1*) and Panx1 mRNA expression in cultured astrocytes illustrating the efficacy of siRNA-induced gene silencing. Scr, scrambled siRNA. *Significant reduction of expression, *p* < 0.05. ***C***, Representative examples of mechanosensory [Ca^2+^]_i_ responses recorded in brainstem astrocytes illustrating the effects of connexin channel inhibitor NPPB (200 μm), Cx43 inhibitory peptide Gap26 (100 μm), Cx43 knock-down using Gja1 siRNA, Panx1 inhibitory peptide ^10^Panx (100 μm), TRPV4 channel blocker RN1734 (10 μm), P2Y_1_ receptor antagonist MRS2179 (3 μm), and IP3 receptor inhibitor Xestospongin C (XeC; 1 μm). Traces depict individual (gray) and averaged (black/blue) changes in fluorescence of Ca^2+^-sensitive genetically encoded sensor *Case12* recorded in 6–15 individual astrocytes in culture. In the experiments involving Cx43 knock-down with Gja1 siRNA, ATP was applied at the end of the recordings to confirm cell viability. Time bars: 150 s. ***D***, Summary data illustrating the effects of blocking Panx1 channels, connexin channels, Cx43, TRPV4 channels, P2Y_1_ receptors, ryanodine receptors, IP3 receptors, and PLC on mechanosensory Ca^2+^ responses in brainstem astrocytes. Rya, inhibitory ryanodine (10 μm); 2-APB, 2-aminoethoxydiphenylborane (30 μm). Data points show averaged peak magnitude of the second [Ca^2+^]_i_ response (expressed as the percentage of the first response) recorded in 6–20 individual astrocytes in separate experiments (*n* = 3–6 cultures). ***E***, Summary data illustrating increases in concentration of ATP in the incubation media after the mechanical stimulation of cultured astrocytes in the absence and presence of Gap26 (100 μm). Data points depict differences in ATP concentration before and after the mechanical stimulation, recorded in separate experiments (*n* = 8 cultures in each group); *p* values, Kruskal–Wallis ANOVA.

Two protocols of mechanical stimulation of astrocytes in culture were used in this study. In one protocol, a single astrocyte in the center of the field of view was approached with a blunt tip glass pipette filled with HBSS. The cell was mechanically stimulated by pressure ejection of HBSS (2 psi, 100-ms pulse; Pneumatic PicoPump PV820, WPI) from a distance of ∼20–100 µm from the cell membrane (Fig. 1*A*). In the second protocol, magnetite (iron oxide, Fe_3_O_4_) particles were applied to astrocytes in culture and cells were mechanically stimulated by application of the magnetic field. Magnetite particles (<5 µm; Sigma catalog #310069) were first pretreated in a solution containing 0.6 mg ml^−1^ collagen for 1 h at 37°C followed by 3 rinses in PBS. Particles were then sonicated to eliminate clumping and added to the cell cultures at a concentration 0.2 mg ml^−1^ for 1 h followed by two washes with HBSS to remove unattached particles. Mechanical stimulation was applied by an electromagnet which generated magnetic field over the recording chamber (Fig. 2*A*).

#### Analysis of the mechanosensory transduction mechanism

To investigate the mechanisms underlying the mechanosensory Ca^2+^ responses of brainstem astrocytes, the stimuli were applied in the absence and presence of pharmacological agents or after application of small interfering RNA (siRNA) to block the hypothesised mechanosensory transduction pathways. Carbenoxolone (CBX) in 10 μm concentration (Bruzzone et al., 2005), probenecid (1 mm; Silverman et al., 2009), or mimetic peptide ^10^Panx (100 μm) were used to inhibit Panx1 hemichannels. CBX in 100 μm concentration, 5-nitro-2-(3-phenylpropylamino)-benzoic acid (NPPB; 200 μm), or proadifen (100 μm) were applied to block connexin hemichannels (Huckstepp et al., 2010). Mimetic peptide Gap26 (100 μm) was used to block connexin 43 (Cx43) channels (Evans et al., 2012). RN1734 (10 and 100 μm) or HC-067047 (10 μm) were applied to inhibit TRPV4 channels. P2Y_1_ receptors were blocked with MRS2179 (3 μm). Ryanodine receptors were blocked with inhibitory ryanodine (Rya; 10 μm). Phospholipase C (PLC) activity was inhibited with U73122 (10 μm). IP3 receptors were inhibited with Xestospongin C (1 μm) or 2-aminoethoxydiphenylborane (30 μm). EGTA (1 mm) was used to chelate Ca^2+^. All drugs were obtained from Tocris Bioscience.

Panx1 siRNA (50 pm; Thermo Fisher Scientific) or Gja1 (Cx43) siRNA (50 pm; Thermo Fisher Scientific) were applied to astroglial cultures for 24 h to knock-down the expression of Panx1 or Cx43 channels, respectively (Iglesias et al., 2009). Quantitative real-time PCR (RT-qPCR) was used to determine the efficacy of gene knock-down. RNA was purified using the RNeasy Micro kit (QIAGEN) and reverse transcribed using the QuantiTect Reverse Transciption kit (QIAGEN) as per manufacturer's protocol. PCRs were performed in duplicates using the TaqMan Universal Master Mix II using the TaqMan assays as the detection method and an Applied Biosystems 7500 RT-PCR system (Applied Biosystems). Relative Panx1 (Rn01447976_m1; Thermo Fisher Scientific) and Cx43 (Rn01433957_ml; Thermo Fisher Scientific) gene expression values were calculated using the comparative ΔΔCt method normalized to the expression of GAPDH (Rn01775763_g1, 174-bp amplicon length, Life Technologies).

#### Quantification of ATP release induced by mechanical stimulation of astrocytes

Magnetite particles were added to astrocyte cell cultures followed by two washes with HBSS to remove the unattached particles. After 30 min, a sample (80 µl) of the incubation media was collected for the assessment of basal ATP release. Mechanical stimulation was next applied by an electromagnet which generated magnetic field over the recording chamber and the second sample of the media was collected for the measurement of mechanosensory ATP release. Mechanical stimulation was applied in the absence or presence of Gap26 (100 μm). Concentration of ATP in samples was determined using luciferin-luciferase assay (CellTiter-Glo, Promega Corporation). The bioluminescence was recorded using an IVIS Spectrum imaging system (PerkinElmer) and the photon count was converted to [ATP] using a standard 5-point (0–20 nm) calibration curve.

#### Proximity ligation assay (PLA)

**Figure 3.**
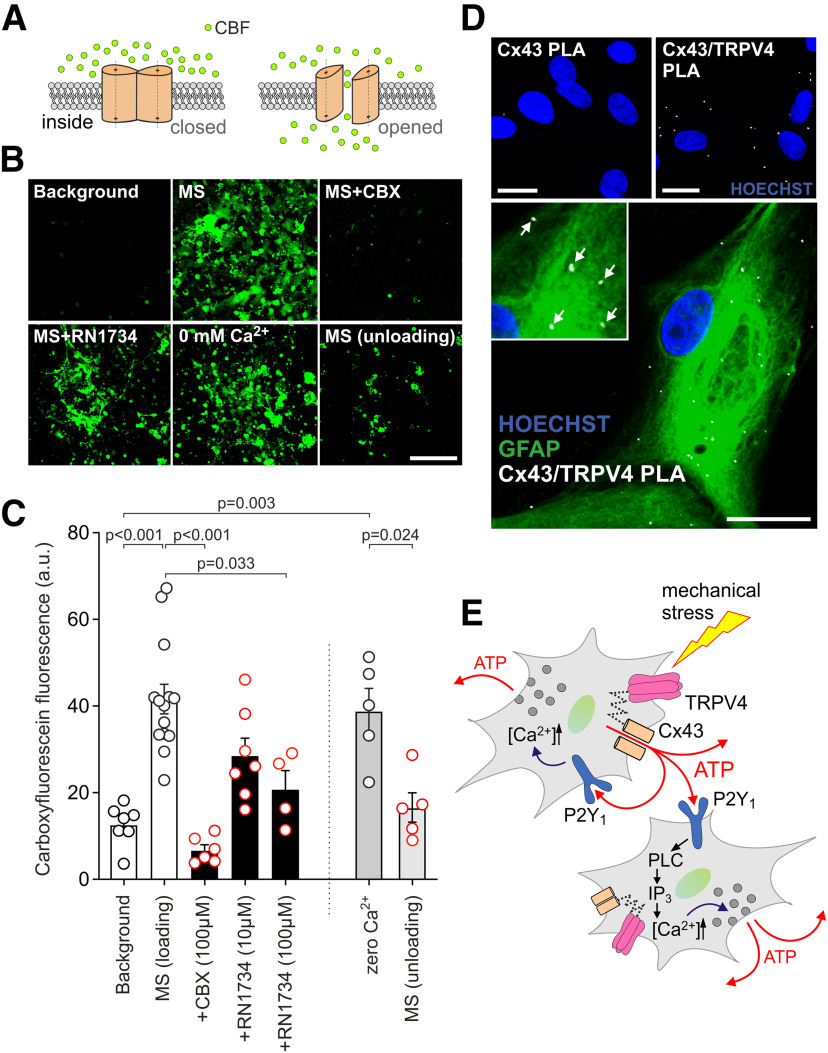
Opening of connexin hemichannels in response to mechanical stimulation. Cx43 and TRPV4 channel interaction in astrocytes. ***A***, Assessment of hemichannel open probability by carboxyfluorescein (CBF) dye loading. Connexin hemichannels are permeable to CBF (376 Da) and in an open state can act as conduits of CBF transport across the membrane in accord with the concentration gradient of the dye. ***B***, ***C***, Representative images and summary data of CBF fluorescence in cultured astrocytes coated with magnetite particles illustrating background dye loading and intracellular CBF accumulation (loading) in response to mechanical stimulation by application of the magnetic field in the absence and presence of CBX and RN1734. In separate experiments, astrocytes were exposed to 0 mm extracellular [Ca^2+^] to increase open probability of connexin hemichannels and promote CBF loading. Then, Ca^2+^ was added to the incubation media to close the channels, extracellular CBF was removed by washing and mechanical stimulation applied, resulting in CBF unloading. Data points show CBF fluorescence in all astrocytes in the field of view, recorded in separate experiments (*n* = 4–14 cultures). Scale bar: 100 µm. ***D***, Micrographs of PLA in brainstem astrocytes showing interaction between Cx43 and TRPV4 channels. Puncta reveal positive PLA signals, indicating that Cx43 and TRPV4 proteins are in proximity (<40 nm). First panel shows the negative control for the PLA assay, performed in the absence of anti-TRPV4 antibody. Hoechst staining was used to visualize the nuclei. Scale bars: 20 µm. ***E***, Schematic diagram of mechanosensory signaling in astrocytes mediated by interaction of TRPV4 channels and Cx43 hemichannels, leading to the release of ATP, which propagates/amplifies astroglial Ca^2+^ excitation via P2Y_1_ receptor activation and Ca^2+^ recruitment from the intracellular stores.

*In situ* PLA (Söderberg et al., 2008) was used to determine the interaction between Cx43 and TRPV4 in astrocytes. In this assay, the two proteins of interest are targeted with primary antibodies raised in different species and then with secondary antibodies conjugated to short DNA oligonucleotides (PLA probes + and –). Only if both PLA probes (and therefore the two proteins) are in proximity (<40 nm), a hybridizing connector oligo joins them, and ligase enzyme forms a closed circular DNA molecule which is amplified by DNA polymerase. Finally, fluorochrome-labeled oligos bind to the amplicon, allowing visualization of protein interactions as discrete spots (PLA fluorescent signals). Astrocytes plated on coverslips were fixed in 4% formaldehyde for 15 min. After fixation, the cells were washed with PBS, incubated for 1 h at room temperature in PBS containing 0.2% Triton X-100 and 10% donkey serum, and then incubated overnight at 4°C with anti-Cx43 mouse monoclonal antibody (1:250; Millipore #MAB3067) and anti-TRPV4 rabbit polyclonal antibody (1:250; Abcam ab94868). Duolink-PLA Red (Sigma) was then performed according to the manufacturer instructions. Separately, for immunohistochemical validation of the antibodies, the cells were incubated for 1 h with the corresponding fluorophore-conjugated secondary antibodies (anti-mouse Alexa Fluor 488 and anti-rabbit Alexa Fluor 647). After the completion of the PLA amplification step, cell nuclei were stained with Hoechst dye, and samples were incubated for 3 h with Alexa Fluor 488-labeled anti-GFAP antibody (1:100; Abcam ab194324) to reveal the cell morphology. Omission of TRPV4 antibody was used as a negative control. Images were acquired using a Zeiss 710 VIS CLMS confocal microscope equipped with a META detection system and a 40× oil immersion objective. PLA fluorescent signals (white dots) within the astrocytes were detected only when both antibodies were present (Fig. 3*D*).

#### Astrocyte-specific Cx43 gene knock-out mice

Astrocyte-specific Cx43 knock-out mice were generated as previously described (Clasadonte et al., 2017). Briefly, homozygous floxed Cx43 mice (Cx43^flox/flox^), in which Exon 2 of Cx43 allele is flanked by two LoxP sites, were bred to human glial fibrillary acidic protein (hGFAP)-Cre mice, obtained by inserting a DNA fragment encoding the Cre recombinase into an expression cassette containing a 2.2-kb human GFAP promoter, *gfa2*. Experimental animals were generated by crossing homozygous Cx43^flox/flox^ mice with Cre-positive mice (Cx43^flox/flox^: GFAP^Cre+^). Control animals were produced by crossing homozygous Cx43^flox/flox^ animals with Cre-negative mice (Cx43^flox/flox^: GFAP^Cre–^). PCR genotyping from tail biopsy DNA was performed by using the following primers: for floxed Cx43, corresponding to a 580-bp band, (forward) 5′-CTTTGACTCTGATTACAGAGCTTAA-3′ and (reverse) 5′-GTCTCACTGTTACTTAACAGCTTGA-3′; for hGFAP-Cre, giving a 500-bp band, (forward) 5′-GGTCGATGCAACGAGTGATGAGG-3′ and (reverse) 5′-GCTAAGTGCCTTCTCTACACCTGCG-3′.

Cx43 deletion in brainstem astrocytes was confirmed by Western blot analysis and immunohistochemical detection of Cx43 protein expression. Mice were terminally anesthetized with isofluorane and transcardially perfused with saline, followed by ice-cold 4% paraformaldehyde (PFA) in PBS (pH 7.4). The brains were removed, postfixed in the same solution for 12 h, and cryoprotected in 30% sucrose for 24 h. The brainstems were isolated and sliced (30-µm coronal sections). Proteins from the PFA-fixed tissue were extracted as described in detail in Guo et al. (2012). Protein quantification was performed using a Pierce BCA Protein Assay kit (Thermo Fisher Scientific). Cx43 was immunodetected using rabbit anti-Cx43 antibody (1:1000; Cell Signaling) followed by anti-rabbit IgG-HRP (1:10,000; Thermo Fisher Scientific). Proteins were electrophoretically separated in SDS-PAGE gels and transferred to Immobilon-P polyvinylidene fluoride membranes (Millipore). After antibody labeling, immunoreactivity was revealed using Western Lightning Plus-ECL (PerkinElmer) and imaged using a Fujifilm LAS4000 system with ImageQuant software. Densitometry was used to calculate the level of Cx43 expression normalized to the expression of β-actin (mouse anti-β-actin antibody; 1:10,000; Sigma; followed by anti-mouse IgG-HRP; 1:10,000; Thermo Fisher Scientific) to control protein loading. A SeeBlue Plus2 Pre-Stained standard (Life Technologies) was used to estimate protein sizes.

Separately, the expression of Cx43 in the brainstems of astrocyte-specific Cx43 and control mice was assessed by immunostaining. Brainstem sections were incubated overnight (at 4°C) with rabbit anti-Cx43 antibody (1:1000; Cell Signaling) and chicken anti-GFAP antibody (1:500, Abcam), followed by incubation with fluorochrome-conjugated goat anti-rabbit Alexa Fluor 488 and goat anti-chicken Alexa Fluor 546 secondary antibodies (each at 1:1000 dilution). Sections were mounted with Vectashield antifade mounting medium containing DAPI. Images of the entire brainstem sections were automatically acquired using an epifluorescence microscope (Keyence BZ-X700) with a 20× objective. High-magnification images were acquired using a confocal microscope (Nikon A1) with a 40× objective.

#### *In vivo* experiments

**Figure 4.**
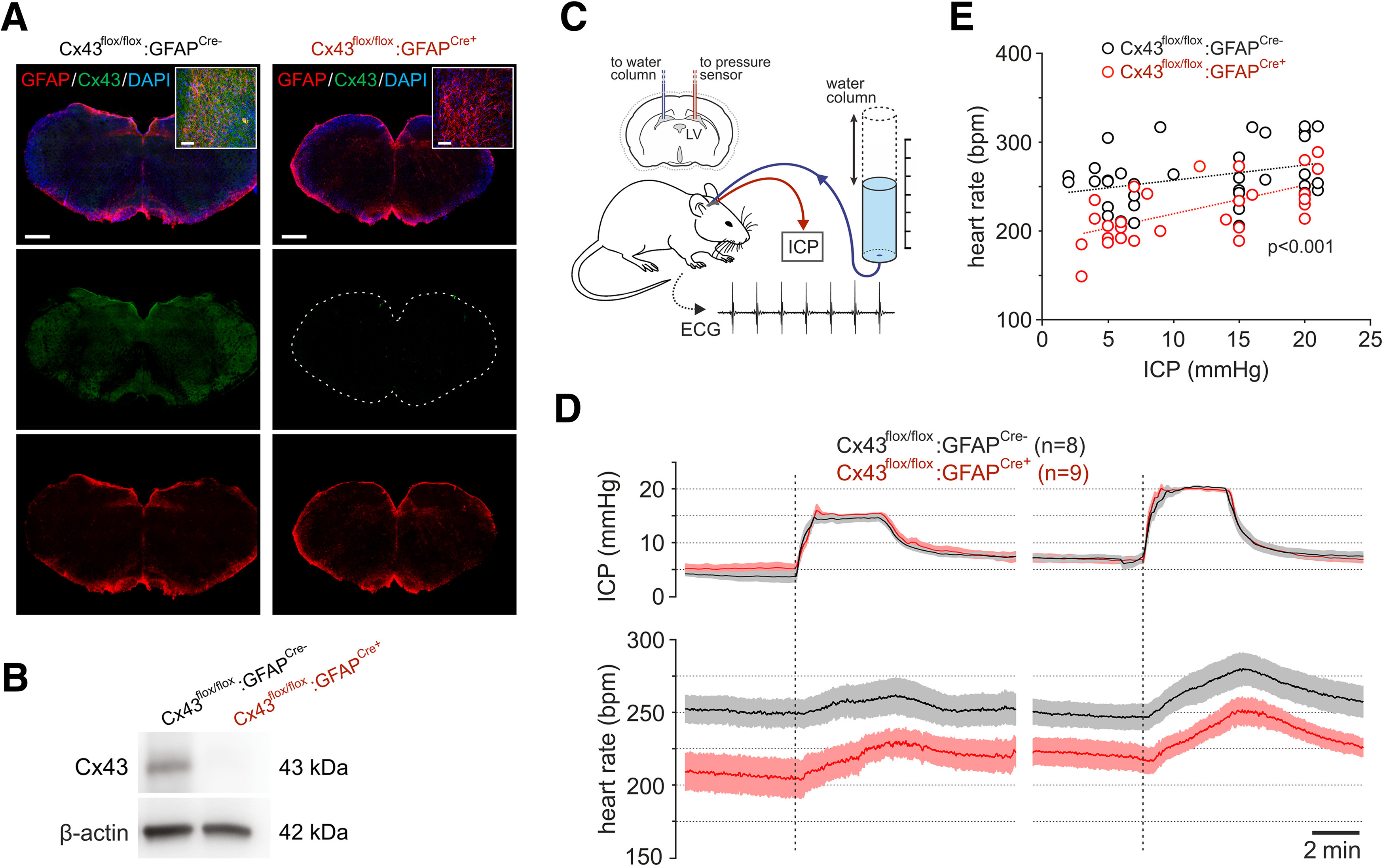
Resting heart rate and heart rate responses to acute changes in brain perfusion in astrocyte-specific Cx43 knock-out mice. ***A***, Representative immunofluorescence micrographs of Cx43 (green) and GFAP (red) expression in the brainstems of Cx43^flox/flox^:GFAP^Cre–^ and Cx43^flox/flox^:GFAP^Cre+^ mice, confirming effective deletion of Cx43 in this model. Scale bars: 500 µm. Insets, *Z*-stack images taken at higher magnification. Scale bars: 50 µm. ***B***, Representative immunoblot showing Cx43 expression in the brainstems of Cx43^flox/flox^:GFAP^Cre–^ and Cx43^flox/flox^:GFAP^Cre+^ mice. ***C***, Diagram of the experimental setup in mice instrumented for the recordings of ICP via a cannula implanted into the left lateral cerebral ventricle (LV), and heart rate (ECG in Lead II configuration). Cerebral perfusion pressure was acutely decreased by raising the ICP using a water column connected via a saline-filled mini-catheter to a cannula placed in the right lateral cerebral ventricle. ***D***, Summary data (mean ± SEM) illustrating resting heart rate and heart rate responses to acute changes in ICP in astrocyte-specific Cx43 knock-out (Cx43^flox/flox^: GFAP^Cre+^) and control (Cx43^flox/flox^: GFAP^Cre–^) mice. ***E***, Individual heart rate data taken from the recordings illustrated in ***D*** and plotted against the corresponding values of ICP showing significantly lower heart rates in astrocyte-specific Cx43 knock-out mice at different levels of ICP; *p* value, Kolmogorov–Smirnov test *D* statistic.

Cx43^flox/flox^: GFAP^Cre+^ (knock-out, *n* = 9) and Cx43^flox/flox^: GFAP^Cre–^ (control, *n* = 9) mice (four to six months old) of both sexes were anesthetized with ketamine 100 mg kg^−1^ and xylazine 10 mg kg^−1^. The depth of anesthesia was monitored using the stability of heart rate and lack of flexor responses to a paw pinch. Supplemental anesthesia was given as required. Body temperature was maintained at ∼37.0°C using a servo‐controlled heating pad. An electrocardiogram (ECG) was recorded using needle electrodes placed subcutaneously in a Lead II configuration. The animal was placed in a stereotaxic apparatus. The left lateral cerebral ventricle was cannulated and connected via a saline-filled mini-catheter to a pressure transducer to record changes in intracranial pressure (ICP; Fig. 4*A*). Correct positioning of the cannula was confirmed by observing cardiac pulse-related small oscillations of ICP. The right lateral cerebral ventricle was cannulated and connected via saline-filled mini-catheter to a “water column” (Fig. 4*A*). Cannulae were secured in place with cyanoacrylate adhesive to ensure a hermetic seal. Considering that the resting cardiac vagal activity in mice is very low (Gehrmann et al., 2000), the heart rate was used as a measure of central cardiac sympathetic drive. As cerebral perfusion pressure is determined by the difference between the mean arterial blood pressure and ICP, experimental decreases in brain perfusion were induced by changing the vertical position of the water column (relative to the surface of the brain) to increase the ICP (Marina et al., 2020). These experiments and the initial data analysis were performed by the investigator blinded to the genotype of the experimental animals. One animal in the Cx43^flox/flox^: GFAP^Cre–^ group was severely arrhythmic during the course of the experiment and the recorded data were excluded from the analysis.

#### Statistical analysis

Imaging data were analyzed using Origin 8.5 software. Physiologic data were acquired using a Power1401 analog to digital interface and analyzed offline using *Spike2* software (Cambridge Electronic Design). Cellular Ca^2+^ responses to mechanical stimuli in the absence and presence of test drugs/treatments, and heart rate responses in Cx43 knock-out and control mice were compared by Kruskal–Wallis ANOVA, two-way ANOVA, or Kolmogorov–Smirnov test *D* statistic, as indicated. For the analysis of RT-qPCR data, the intervals of confidence (95% IC) were obtained by applying the general formula for the propagation of errors to the initial SDs of the duplicates measured for each sample. The data are reported as individual data and/or mean ± SEM.

## Results

To study the mechanosensory signaling in brainstem astrocytes we applied two methods of controlled mechanical stimulation of these cells in culture (Figs. 1*A*, 2*A*). Robust and reproducible Ca^2+^ responses were evoked in individual astrocytes when mechanical stimulation was applied by timed ejections of extracellular media by pressurization of a patch pipette positioned close to the cell membrane (Fig. 1*B*,*C*). It was found that mechanical stimulation-induced Ca^2+^ signals in individual brainstem astrocytes were markedly reduced by pharmacological inhibition of distinct membrane targets: connexin/pannexin channels and gap junctions with CBX (100 μm), TRPV4 channels with RN1734 or P2Y_1_ receptors with MRS2179 (Fig. 1*C*,*D*).

For detailed pharmacological analysis of the mechanosensory transduction mechanism, in the next experiments we coated cultured astrocytes with magnetite particles and applied the magnetic force for mechanical stimulation of the cells (Fig. 2*A*). Using this method of mechanical stimulation, we next found that mechanosensory Ca^2+^ responses in brainstem astrocytes were significantly reduced by the pharmacological agents that inhibit connexin channels (CBX, NPPB, and proadifen; Fig. 2*C*,*D*), and completely abolished by Cx43 blocking peptide Gap26 (Fig. 2*C*,*D*), or in conditions of Cx43 gene knock-down using Gja1 siRNA (Fig. 2*B–D*). Similar approaches (pharmacological, blockade with the mimetic peptide and gene knock-out) applied to inhibit Panx1 had no effect on mechanosensory Ca^2+^ responses in astrocytes (Fig. 2*B–D*).

It was also found that Ca^2+^ responses in astrocytes induced by mechanical stimulation were effectively abolished by pharmacological blockade of Ca^2+^-permeable TRPV4 channels with RN1734 or HC-067047 (Fig. 2*C*,*D*). However, in Ca^2+^ free media (+0.5 mm EGTA), mechanical stimulation still evoked [Ca^2+^]_i_ elevations in astrocytes (Fig. 2*D*), indicative of Ca^2+^ recruitment from the intracellular stores. Indeed, mechanosensory Ca^2+^ responses in astrocytes were abolished by inhibition of PLC activity with U73122 (Fig. 2*D*), or blockade of IP3 receptors with Xestospongin C or 2-APB (Fig. 2*C*,*D*). Astroglial Ca^2+^ responses to mechanical stimulation were also abolished in conditions of P2Y_1_ receptor blockade with MRS2179 (Fig. 2*C*,*D*), suggesting that the release of ATP is ultimately responsible for the mechanosensory Ca^2+^ responses in astrocytes.

Measurements of changes in ATP concentration in the incubation media before and after the mechanical stimulation of astrocytes support this conclusion. Application of the magnetic force to astrocytes coated with magnetite particles increased the concentration of ATP in the media by 3.3 ± 0.7 nm (from 5.7 ± 0.6 to 9.0 ± 1.1 nm; *n* = 8, *p* = 0.002; Fig. 2*E*). In the presence of Gap26, the mechanosensory ATP release was reduced by ∼60% (increase by 1.3 ± 0.5 nm; from 5.6 ± 0.7 to 6.9 ± 1.0 nm; *n* = 8; *p* = 0.04 compared with the control release; Fig. 2*E*).

Release via hemichannels is one of the potential mechanisms of ATP secretion by astrocytes (Lohman and Isakson, 2014). Connexin hemichannels in an opened state are permeable to carboxyfluorescein (Huckstepp et al., 2010; Meigh et al., 2013; Fig. 3*A*). In the presence of carboxyfluorescein in the incubation media, mechanical stimulation of astrocytes facilitated intracellular accumulation of the dye (Fig. 3*B*,*C*), indicating increased open probability of membrane channels. Mechanical stimulation-induced dye accumulation was prevented by CBX (Fig. 3*B*,*C*) and inhibited by TRPV4 blocker RN1734 (Fig. 3*B*,*C*). In separate experiments, astrocytes were exposed to 0 mm extracellular [Ca^2+^], known to increase open probability of connexin hemichannels and promote carboxyfluorescein dye loading (Hadjihambi et al., 2017; Fig. 3*B*,*C*). Then Ca^2+^ (2 mm) was added to the incubation media to close the channels, and extracellular carboxyfluorescein was removed by washing. In these conditions, application of mechanical stimulation reduced intracellular carboxyfluorescein fluorescence (unloading; Fig. 3*B*,*C*), indicating washout of the dye via the membrane channels gated by the mechanical stimuli. PLA demonstrated interaction between Cx43 and TRPV4 in astrocytes (Fig. 3*D*).

We next determined the significance of the identified mechanism of astroglial mechanosensitivity for the operation of intracranial baroreflex that mediates the sympathetic and cardiovascular responses to changes in brain perfusion (Marina et al., 2020). There is evidence that these responses are triggered or facilitated by brainstem astrocytes that sense decreases in cerebral perfusion pressure and activate neighboring presympathetic neurons to increase systemic arterial blood pressure and heart rate (Marina et al., 2020). Since Cx43 was identified as a critical component of the mechanosensory transduction mechanism in brainstem astrocytes, we hypothesized that deletion of Cx43 specifically in astrocytes would have an impact on central nervous control of heart rate. It was found that in mice with constitutive deletion of Cx43 in astrocytes (Fig. 4*A*,*B*), the resting heart rate was markedly (by 18%; *p* < 0.001) lower compared with the control animals (Fig. 4*D*,*E*). Although the responses to decreases in cerebral perfusion (induced by experimentally-induced increases in ICP) were of similar magnitudes (Fig. 4*D*), heart rates recorded in astrocyte-specific Cx43 knock-out mice were lower across the whole range of ICPs tested (2–22 mmHg; Fig. 4*E*).

## Discussion

Sensitivity to mechanical stimuli is a well-known feature of astrocytes (Moshayedi et al., 2010). However, until very recently the functional significance of astroglial mechanosensitivity remained unclear, as in healthy conditions the mechanical forces experienced by the brain tissue cushioned by the cerebrospinal fluid within the cranium would be expected to be negligible. Kim and colleagues (Kim et al., 2015) first reported mechanosensory Ca^2+^ signals recorded in brain slices from perivascular astrocytes in response to experimentally-induced increases in pressure/flow in the associated parenchymal arteriole, presumably leading to increases in vessel diameter and stretch of the tight astroglial end-feet. A recent *in vivo* study (Marina et al., 2020) demonstrated that astrocytes display immediate Ca^2+^ responses to acute drops in cerebral perfusion induced by increases in ICP by 10–15 mmHg, known to occur physiologically in response to acute postural changes, for example (Petersen et al., 2016). Interestingly, responses of cortical astrocytes to changes in ICP, with Ca^2+^ peaks at the stimulus onset and offset (Marina et al., 2020), share a striking resemblance with the response profiles of rapidly adapting peripheral mechanosensory neurons (Lumpkin et al., 2010), although the responses of astrocytes and neurons develop over the different timescales.

To study the mechanosensory signaling in astrocytes we applied two methods of controlled mechanical stimulation of these cells in culture. Stimulation was applied by timed ejections of extracellular media by pressurization of a patch pipette positioned close to the astrocyte membrane or by coating astrocytes with magnetite particles and application of the magnetic field. As elevated pressure in a closed system leads to a stretch of cell membranes within that system (as discussed by Beckel et al., 2014), we consider these *in vitro* models appropriate for the purpose of studying the mechanosensory transduction mechanisms underlying the function of astrocytes as intracranial baroreceptors. Pharmacological analysis of the responses induced by two modes of mechanical stimulation *in vitro* suggested activation of the same signaling mechanism involving functional interaction of mechanosensitive TRPV4 channels and Cx43 hemichannels, leading to the Cx43-mediated release of ATP. ATP propagates/amplifies astroglial Ca^2+^ excitation via P2Y_1_ receptor activation and Ca^2+^ recruitment from the intracellular stores (Fig. 3*E*).

Earlier studies of Dahl and colleagues conducted in *Xenopus* oocytes first demonstrated mechanosensory release of ATP via Panx1 (Bao et al., 2004a) and connexin 46 hemichannels (Bao et al., 2004b). More recently, release of ATP through Panx1 channels was demonstrated in astrocytes of the optic nerve head in response to stretch and swelling (Beckel et al., 2014). Other studies addressed various aspects of mechanosensory signaling in brain astrocytes. In a study conducted in cultured cortical astrocytes, Darby et al. (2003) demonstrated that cell swelling causes ATP release via multidrug resistance protein transport. Bowser and Khakh (2007) reported that vesicular release of ATP acting at P2Y_1_ receptors propagates mechanical stimulation induced Ca^2+^ excitation in cultured hippocampal astrocytes. A similar mechanism of mechanosensory Ca^2+^ signal propagation was also described in retinal Müller cells (Agte et al., 2017). In general, the results reported here are in agreement with the existing data, yet we found no evidence that Panx1 channels mediate mechanosensory Ca^2+^ responses in brainstem astrocytes, as these responses were unaffected by pharmacological blockade or genetic Panx1 silencing.

Under the same experimental conditions, mechanosensory Ca^2+^ responses in astrocytes were fully blocked by mimetic peptide Gap26, which is a highly selective inhibitor of Cx43 (Evans et al., 2012), or by Cx43 gene knock-down with Gja1 siRNA. Enhanced carboxyfluorescein dye loading of astrocytes directly demonstrated opening of CBX-sensitive membrane channels in response to mechanical stimulation. These data strongly suggest that opening of Cx43 hemichannels is the key central event underlying sensitivity of astrocytes to mechanical stimuli. This, however, also requires TRPV4 channels, as mechanosensory Ca^2+^ responses in astrocytes could also be effectively inhibited by pharmacological blockade of these channels. TRPV4 channels are mechanosensitive Ca^2+^-permeable channels with diverse functions (White et al., 2016). TRPV4 channels are expressed in astrocytes and contribute to Ca^2+^ oscillations in the end-feet during periods of increased neuronal activity (Dunn et al., 2013). Although TRPV4 channels had been shown to respond to mechanical stimuli applied to the cell membrane in several organ tissues, it remains unknown how the mechanical force (cell/membrane stretch) gates these channels (White et al., 2016). In our experiments, we found no evidence of direct TRPV4 channel gating, as mechanosensory Ca^2+^ responses in astrocytes were unaffected in the absence of extracellular Ca^2+^. On the other hand, pharmacological blockade of TRPV4 partially inhibited mechanical stimulation-induced carboxyfluorescein dye loading, suggesting that TRPV4 channels contribute to mechanical “gating” of Cx43 hemichannels. That activation of TRPV4 channels can trigger connexin hemichannel-mediated release of ATP was shown previously in the epithelial cells of the lens and the esophagus (Ueda et al., 2011; Shahidullah et al., 2012), although the exact mechanisms of how these channels may interact remain unknown. In this study, PLA confirmed the possibility of direct functional interaction between TRPV4 and Cx43 in astrocytes. Collectively the data obtained allow us to hypothesize that membrane stretch causes TRPV4 channel activation and conformational change that is imparted on Cx43 hemichannels increasing their open probability leading to the release of ATP (Fig. 3*E*).

ATP is the key signaling molecule that mediates communications between astrocytes and neurons (Araque et al., 2014). There is also evidence that astrocytes signal to neighboring neurons via the release of lactate (Tang et al., 2014) and that connexin hemichannels may function as conduits of lactate transport across the membrane (Karagiannis et al., 2016; Clasadonte et al., 2017). ATP and lactate actions in the ventral regions of the brainstem have strong sympathoexcitatory effects and increase the vasomotor and cardiac sympathetic activities leading to the increases in the arterial blood pressure and heart rate (Horiuchi et al., 1999; Marina et al., 2013, 2015). In this study, we found that astrocyte-specific Cx43 knock-out mice display significantly lower heart rates at any given level of cerebral perfusion which was experimentally altered by changes in ICP. As cardiac vagal activity in mice kept under standard laboratory conditions is weak (Gehrmann et al., 2000), lower heart rates in astrocyte-specific Cx43 knock-out mice most likely reflect lower levels of cardiac sympathetic activity. This conclusion is consistent with the hypothesis of Cx43-mediated release of ATP (and presumably lactate also) by the astrocytes having an excitatory action on the brainstem sympathetic control circuits. Yet, the profile and the magnitude of heart rate responses to acute decreases in brain perfusion were not affected by Cx43 deletion. As mature astrocytes express several other members of the connexin family (Cx30 is another notable astroglial connexin; Nagy and Rash, 2000), there is a possibility of functional compensation for Cx43 loss by other connexins in this knock-out mouse model.

In conclusion, this study describes the mechanisms underlying responses of brainstem astrocytes to mechanical stimuli. The data obtained suggest that mechanosensory transduction in astrocytes relies on functional interaction between TRPV4 and Cx43 channels and leads to Cx43 hemichannel-mediated release of ATP. The heart rate phenotype of mice with astrocyte-specific genetic deletion of Cx43 is consistent with the hypothesis of mechanosensory connexin hemichannel-mediated release of signaling molecules by astrocytes having an excitatory action on the brainstem sympathetic control circuits.
